# The Effects of CB2R Activation on Inflammatory Pathways in Dermatomyositis

**DOI:** 10.3390/biomedicines14061296

**Published:** 2026-06-07

**Authors:** Rohan Dhiman, Ahmed Eldaboush, Navin Vijayarangan, Darae Kang, Nilesh Kodali, DeAnna Diaz, Caroline Stone, Rui Feng, Victoria P. Werth

**Affiliations:** 1 Corporal Michael J. Crescenz VA Medical Center, Philadelphia, PA 19104, USA; rohandh@sas.upenn.edu (R.D.);; 2 Department of Dermatology, University of Pennsylvania Perelman School of Medicine, Philadelphia, PA 19104, USA; 3 Department of Biostatistics, University of Pennsylvania, Perelman School of Medicine, Philadelphia, PA 19104, USA

**Keywords:** cannabinoid receptor 2 (CB2R), peripheral blood mononuclear cells (PBMCs), dermatomyositis (DM)

## Abstract

**Background/Objectives:** Dermatomyositis is an autoimmune disease with heterogeneous symptoms and many potential drivers. Nonpsychoactive cannabinoids have shown promise in treating some subtypes of DM; however, the reasons behind this were unclear. In this project, we tested the effects of CB2R activation on PBMCs from amyopathic and classic DM patients to determine its anti-inflammatory effects on pathways biologically relevant to DM. **Methods:** We determined the % CB2R positivity and intracellular cytokines in PBMCs from amyopathic DM and classic DM patients. CB2R positivity was determined by analyzing patient PBMCs via flow cytometry. PBMCs were stimulated by dsRNA for RIG1, dsDNA for cGAS, LPS for TLR4, and LPS/ATP for NLRP3, with and without CB2R pretreatment, and IFNβ, IFNγ, p65 NFkB, and pSTING levels were used as markers of pathway activation. The CB2R agonist JWH133 was used to pretreat PBMCs before stimulation. **Results:** Amyopathic DM PBMCS were found to be 101.3% more positive for CB2R compared to classic DM PBMCS (*p* < 0.05). In amyopathic DM PBMCs stimulated by LPS/ATP to target the NLRP3 inflammasome, CB2R activation resulted in a significant reduction in IFNβ MFI for MoDCs (*p* < 0.05) and Macs (*p* < 0.05), with a similar trend observed in cDCs relative to classic DM PBMCS. On the other hand, no difference in IFNβ response to CB2R activation was observed across all cell types investigated between classic and amyopathic DM PBMCs stimulated with LPS only to target TLR4. **Conclusions:** Amyopathic DM PBMCs were significantly more positive for CB2R and had better anti-inflammatory responses to CB2R activation for many inflammatory pathways implicated in DM.

## 1. Introduction

Dermatomyositis (DM) continues to be a challenging disease to manage as current treatments fall short for many patients. Part of the challenge is the heterogeneity across triggers and signs of DM cases [1]. Under the DM umbrella lie subtypes characterized by either their specific symptoms or suspected triggers. Of these subtypes, classic and amyopathic DM are grouped by their symptoms, with classic DM involving both skin and muscle symptoms, while amyopathic DM only involves the skin [2]. This range of subtypes suggests that there may be a variety of inflammatory pathways that lead to common symptoms that characterize DM. Work from our lab has demonstrated that DM can be caused or exacerbated by the herbal supplement spirulina, and that blocking TLR4 inhibits this effect, implicating TLR4 as a potential inflammatory PRR in DM [3]. Our lab found that most pathway markers specifically related to the type I IFN system, such as pSTING, pIRF3, pTBK1, pNFkB, and IFNβ were all increased in DM skin compared to HC skin [4]. We also found that DM EVs stimulate an immune response in peripheral blood mononuclear cells (PBMCs) via cGAS-STING activation [5]. Other studies found that 50% of DM perifascicular myofibers were RIG-1 positive compared to 11% for non-DM, and protein expression of NLRP3 in DM muscle was significantly higher than in controls [6,7]. These distinct PRRs, when activated, ultimately stimulate production of IFNβ and IFNγ. Mounting evidence suggests IFNβ and IFNγ are major drivers of disease in DM, with a study from 2012 using 39 DM skin biopsies showing that IFNβ and IFNγ, but not IFNκ or IFNα mRNA transcript levels, correlated with IFN score [8]. Furthermore, a 2017 study found that DM serum IFNβ levels correlated with skin activity [9]. Supporting this, a recent trial investigating the efficacy of an anti-IFNβ antibody in DM patients found a significant reduction in the activity component of the Cutaneous Dermatomyositis Disease Area and Severity Index (CDASI-A), a validated measure of skin activity in DM, 4 weeks after starting treatment [10]. One of the most promising novel DM treatments is the cannabinoid type 2 receptor (CB2R) agonist Lenabasum. Clinical trials have demonstrated Lenabasum to be more effective than hydroxychloroquine in certain groups of DM patients, while other studies have found subsets of DM patients that do not respond to Lenabasum treatment [11,12]. Specifically, a phase 2 trial of Lenabasum with primarily amyopathic DM patients resulted in a significant decrease in CDASI score, while a phase 3 trial primarily composed of classic DM patients failed to find any benefit, implying that Lenabasum may be more effective in amyopathic compared to classic DM [11,12]. These findings prompted an in vitro investigation of the immune cells modulated by CB2R activation. We found that CB2R was activated by Lenabasum in CD4, MoDC, iMs, pDCs, and CMs in DM PBMCs that responded to Lenabasum treatment [13]. The difference in response to CB2R treatment on a cellular level points to potentially different inflammatory mechanisms between the responders and non-responders. This, coupled with the observation that Lenabasum treatment is more effective in amyopathic vs. classic DM, prompts the question of whether there is a correlation between DM subtype and response to CB2R treatment. Previous in vitro experiments investigating CB2R treatment in DM used PMA/ionomycin and R848 for stimulation; however, these target inflammatory pathways are not biologically relevant to DM [13]. For this reason, to further investigate the effect of CB2R activation in DM leukocytes, we used biologically relevant agents to stimulate the following PRRs: RIG1, cGAS, TLR4, and NLRP3. Given that previous findings show prominent differences in Lenabasum treatment for amyopathic versus classic DM patients, we chose to compare patient PBMCs from these two subtypes. Nonpsychoactive CB2R ligand JWH133 was used for these experiments. As discussed earlier, IFNβ and IFNγ are known drivers of DM and correlate with CDASI severity. Therefore, we chose these cytokines as markers for inflammation intensity. In addition, we also measured p65NFkB and pSTING, as these inflammatory regulators have been found to be upregulated in DM. Finally, we aimed to characterize DM PBMCs for CB2R positivity to determine if expression of this receptor is associated with one DM subtype over another.

## 2. Materials and Methods

### 2.1. Subjects

All patients provided signed consent and ethical approval through the Institutional Review Board at the University of Pennsylvania before participating in the study. The 7 DM patients (4 amyopathic, 3 classic) involved in this study were recruited through the Department of Dermatology at the University of Pennsylvania (Table 1). These patients were diagnosed according to the Sontheimer or EULAR/American College of Rheumatology (ACR) criteria by the PI (Dr. Victoria P. Werth).

### 2.2. PBMC Isolation

In total, 30 mL of blood was collected in six 10 mL heparinized tubes (BD, Franklin Lakes, NJ, USA, #367880), which were then spun at 500 G for 10 min. The plasma was discarded, and the resulting blood was transferred to four 50 mL tubes. A total of 12.5 mL of Ficoll (Millipore Sigma, Burlington, MA, USA, #GE17-1440-02) was added underneath the blood layer for each tube and centrifuged at 500 G for 20 min. The buffy coat was then collected from each tube and combined into one 50 mL tube. These cells were then washed in PBS (Corning^®^, Tewksbury, MA, USA, MT21040CM) and resuspended in 30 mL PBS. In total, 50 μL of this cell suspension was stained with trypan blue (Corning^®^ USA, 25-900-CL) and used at a 1:5 ratio to count the number of PBMCs via a hemocytometer.

### 2.3. Cell Culture

PBMCs destined for CB2R characterization were resuspended in cell culture media {10% fetal bovine serum (Cytiva, Marlborough, MA, USA, #SH30071.03), 1% L-glutamine (Gibco^®M^, Waltham, MA, USA, #25030081), 1% Penicillin-Streptomycin (Gibco^®M^ USA, #15140122) at a concentration of 1 million cells per 1 mL in 100 mm petri dishes until they were stained for flow analysis. PBMCs tend to exhibit significantly lower CB2R expression compared to analogous leukocytes in skin. However, our lab has previously found that supernatant from a fibroblast growth culture can upregulate CB2R expression on PMBCs [14]. For this reason, PBMCs used for stimulation/treatment experiments were resuspended and incubated with supernatant collected from human fibroblast growth media at 1 million cells per 1 mL in 100 mm Petri dishes overnight. These cells were then centrifuged, washed with PBS, resuspended in antibiotic-free cell culture media (10% fetal bovine serum, 1% L-glutamine) at 1 million cells per 1 mL, and plated in 24-well suspension plates for pretreatment and stimulation. CB2R test PBMCs were treated with 3 μM JWH133 (Cayman Chemical USA, Ann Arbor, MI, USA, #10005428) for 2 h, followed by stimulation using the following agents: 200 ng/mL Poly(I:C) (InvivoGen, San Diego, CA, USA, #31852-29-6)/Lyovec^TM^ (InvivoGen USA, #lyec-1) for RIG1, 200 ng/mL Poly(dA:dT) (InvivoGen USA, #86828-69-5)/Lyovec^TM^ for cGAS, 1 μg/mL LPS (SigmaAldrich, Burlington, MA, USA, #L4524-5MG) for TLR4, and 1 μg/mL LPS to prime NLRP3. After 2 h, 10 μg/mL of Brefeldin A (BioLegend, San Diego, CA, USA, #420501) was added, and the PBMCs were allowed to incubate for an additional 3.5 h. Then, 5 mM ATP (InvivoGen USA, #987-65-5) was added to the wells to stimulate NLRP3. At 4 h after adding Brefeldin A, the cells were ready for staining.

### 2.4. Flow Cytometry

PBMCs used for CB2R characterization and stimulation analysis were transferred to FACS tubes and washed with 1 mL staining buffer (2% FBS in PBS) and immediately blocked on ice for 30 min using 4 μL FC block (Invitrogen, Carlsbad, CA, USA, #14-9161-73). In total, 50 μL of BD Horizon Brilliant Stain Buffer (BD USA, #563794) was added afterwards to all tubes. The following surface antibodies were then added to all tubes: BUV563 CD45 (BD USA, #748720), BV 605 CD3 (BioLegend USA, #317322), BUV 496 CD4 (BD USA, #612936), BV786 CD8 (BD USA, #563823), BUV 737 CD56 (eBioscience, Waltham, MA, USA, #1367-0566-42), PE-Cy5 CD19 (eBioscience USA, #15-0199-42), BV480 CD16 (BD USA, #566108), APC-Cy7 HLADR (BD USA, #335796), BUV395 CD14 (BD USA, #563561), BV711 CD123 (Biolegend USA, #306030), and PE-CF594 CD11c (BD USA, #562393). AF700 CB2R (BioTechne Minneapolis, MN, USA, # FAB36551RN-100UG) was added to the CB2R characterization tubes only. They were all then incubated on ice in the dark for 30 min, after which the cells were washed with PBS and fixed with fixation buffer (Biolegend USA, #420801). This was then followed by resuspension in permeabilization buffer (Biolegend USA, #421002). The following antibodies were added to all tubes: PE CD68 (Biolegend USA, #333808) and BUV 661 CD163 (BD USA, #741645). The following antibodies were added to the stimulation tubes only: FITC IFNβ (Bioss Woburn, MA, USA, #BS-0787R-FITC), BV650 IFNγ (BD USA, #563416), PE-Cy7 pNFkB (eBioscience USA, #25-9863-42), and AF647 p-STING (Cell Signaling Technology, Danvers, MA, USA, #43499S). After the cells were allowed to incubate in the dark for 20 min, they were then washed in PBS and resuspended in PBS for analysis. All samples were acquired within 2 days on the BD FACSymphony A3 Cell Analyzer (BD, Franklin Lakes, NJ, USA). Analysis and gating were done using FlowJo v10 (https://www.flowjo.com/).

### 2.5. Statistics

One-tailed Mann–Whitney U tests were used to analyze all findings. All statistics were calculated on GraphPad Prism 9.3.0 (https://www.graphpad.com/). 

## 3. Results

### 3.1. Amyopathic DM PBMCs Express Higher Levels of CB2R Compared to Classic DM

**Figure 1:**
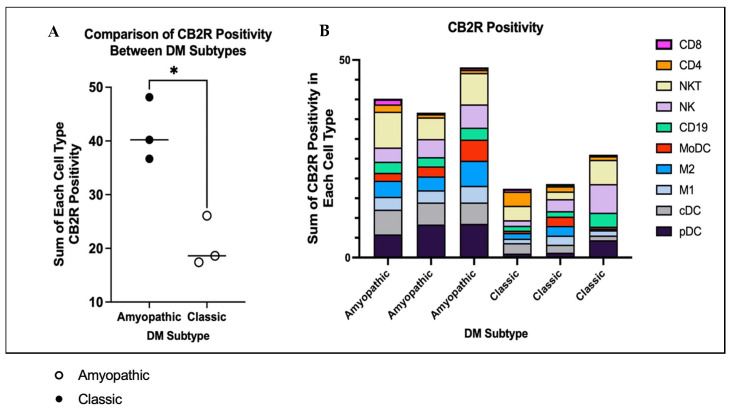
(**A**) PBMCs isolated from amyopathic and classic DM patients were stained for CB2R, and positivity was measured via flow cytometry. The positivity for the following cell types, CD8, CD4, NKT, NK, CD19, MoDC, M2, M1, cDC, and pDC, were summed and compared for each patient sample according to disease subtype. The median of the amyopathic CB2R positivity was found to be significantly more than the classic CB2R positivity (*p* < 0.05). (**B**) CB2R positivity for each cell type for each patient sample was determined via antibody staining and flow cytometry. Each cell type’s CB2R positivity is represented according to the legend on the right. The top 5 cells accounting for the highest CB2R positivity for amyopathic DM are pDC, NKT, cDC, NK, M2 macrophages, and M1 macrophages. The top 5 cells accounting for the highest CB2R positivity for classic DM are NK, NKT, pDC, CD19, and cDC. * *p* < 0.05.

PBMCs were gated for individual cell types, and then CB2R positivity was determined by the percentage of parent positive cells. CB2R positivity data was collected for PBMC CB2R frequency of parent (FoP) for the following cell types: CD4+ T cells, CD8+ T Cells, CD56+ CD3+ NKT cells, CD56 CD3- NK cells, CD19+ B cells, MoDCs, CD68+ CD163+ Macs, CD68+ CD163-Macs, CD11c+ cDCs, and CD123+ pDCs. Amyopathic CD123+ pDCs had a mean CB2R FoP of 7.59%, while classics were 2.21% CB2R positive (*p* < 0.05); amyopathic CD11c+ cDCs had a mean CB2R FoP of 5.76%, while classics were 1.96% CB2R positive (*p* < 0.05); amyopathic CD68+ CD163- Macs had a mean CB2R FoP of 3.51%, while classics were 1.60% CB2R positive (*p* < 0.05); and amyopathic CD68+ CD163+ Macs had a mean CB2R FoP of 4.63%, while classics were 1.41% CB2R positive (*p* < 0.05). In order to compare overall PBMC CB2R positivity between amyopathic and classic DM, the sum of the CB2R FoP of each of the 10 individual cell types was compared between the two DM subtypes. Amyopathic DM PBMCs were 101.3% more positive for CB2R compared to classic DM PBMCS (*p* < 0.05) (Figure 1A). For amyopathic DM PBMCs, the top five cell types accounting for CB2R positivity in decreasing order were the following: pDC, NKT, cDC, NK, and M2 macrophages (Figure 1B). For classic DM PBMCs, the top five cell types accounting for CB2R positivity in decreasing order are the following: NK, NKT, pDC, CD19, and cDC (Figure 1B).

### 3.2. Classic DM PBMCs Have Higher Baseline pSTING Levels Compared to Amyopathic DM PBMCs and Are More Reactive to dsDNA Stimulation While Amyopathic DM PBMCs Appear to Have More NFkB Activity

**Figure 2:**
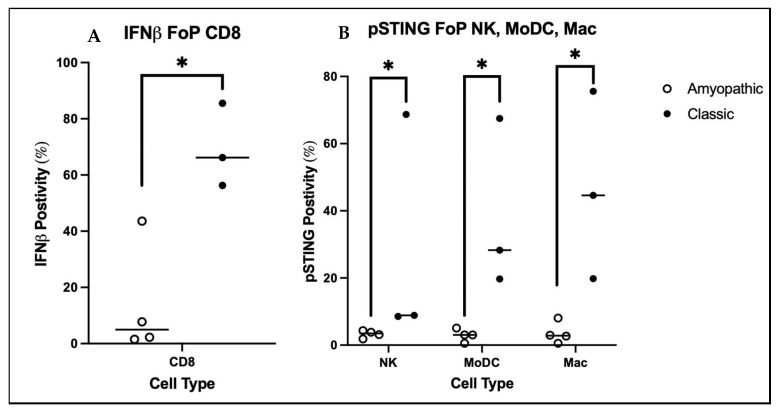
(**A**) Comparison of baseline (unstimulated) IFNβ positivity for amyopathic vs. classic CD8 T cells. Classic CD8 T cells were found to be significantly more positive for IFNβ compared to amyopathic CD8 T cells (*p* < 0.05). (**B**) Comparison of baseline (unstimulated) pSTINGp65 positivity for amyopathic vs. classic NKs, MoDCs and Macs. All three classic cell types were found to be significantly more positive for pSTING compared to amyopathic (*p* < 0.05). * *p* < 0.05.

**Figure 3:**
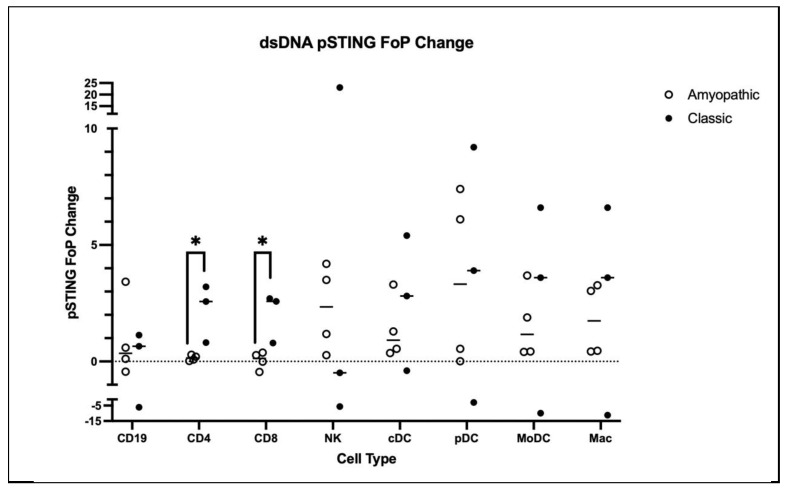
Comparison between amyopathic and classic DM PBMCs for FoP change in pSTING upon dsDNA stimulation. Classic dsDNA CD4 and CD8 T cells show a significant increase in pSTING FoP compared to the FoP change of amyopathic CD4 and CD8 T cells (*p* < 0.05).* *p* < 0.05.

In order to investigate the differences in amyopathic and classic DM PBMCs’ inflammatory pathway activity, baseline levels of IFNβ, IFNγ, p65NFkB, and pSTING were compared. Classic CD8 T cells were significantly more IFNβ-positive compared to amyopathic CD8 T cells (*p* < 0.05) (Figure 2A). Furthermore, classic DM NK, MoDC, and Macs were found to be significantly more positive for pSTING compared to amyopathic DM (*p* < 0.05) (Figure 2B). This higher pSTING activity in classic DM is matched with classic DM CD4 and CD8 T cell pSTING positivity significantly increasing (*p* < 0.05) upon dsDNA stimulation, while there was no significant increase for amyopathic DM CD4 and CD8 T cell pSTING positivity (Figure 3). On the other hand, amyopathic DM CD4 T cells, CD8 T cells, pDCs, MoDCs, and Macs showed a trend of greater p65NFkB FoP increase upon LPS/ATP stimulation, while the same trend was seen for p65NFkB MFI for amyopathic pDCs, MoDCs, and Macs (Supplement Figure S1).

### 3.3. Amyopathic DM LPS/ATP-Stimulated PBMCs Show a Greater Response to CB2R Treatment Compared to Classic PBMCs

**Figure 4:**
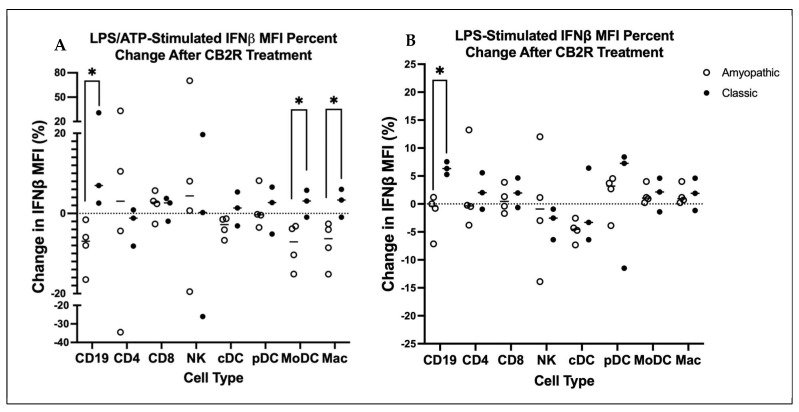
(**A**) Comparison of % change in IFNβ MFI upon CB2R treatment for amyopathic and classic DM PBMCs that were stimulated with LPS/ATP to stimulate TLR4 and NLRP3 formation. LPS/ATP-stimulated amyopathic CD19s, MoDCs and Macs showed a significant decrease in IFNβ MFI compared to classic LPS/ATP-stimulated MoDCs and Macs upon CB2R treatment (*p* < 0.05). (**B**) Comparison of % change in IFNβ MFI upon CB2R treatment for amyopathic and classic DM PBMCs that were stimulated with LPS to stimulate TLR4 only. Only LPS-stimulated amyopathic CD19s showed a significant decrease in IFNβ MFI compared to classic LPS-stimulated CD19s upon CB2R treatment (*p* < 0.05). * *p* < 0.05.

Due to the wide range in baseline values of IFNβ, IFNγ, p65NFkB, and pSTING, the % change in FoP and MFI resulting from CB2R treatment of amyopathic vs. classic DM PBMCs was used for analysis. By first stimulating PBMCs with LPS and then adding 5 mM ATP, the NLRP3 inflammasome is first primed and then activated, resulting in an effect that is mechanistically different from LPS-only stimulation. Amyopathic MoDCs stimulated by LPS/ATP were compared to CB2R agonist-pretreated amyopathic MoDCs stimulated by LPS/ATP. After selecting the IFNβ-positive population of MoDCs, the CB2R-treated amyopathic MoDCs showed a significantly larger decrease in IFNβ MFI compared to classic MoDCs under the same conditions (*p* < 0.05) (Figure 4A). The IFNβ FoP showed a decreasing trend for CB2R-treated amyopathic MoDCs cells. Similarly, LPS/ATP-stimulated CB2R agonist pretreated amyopathic CD68+ Macs and CD19s showed a significantly greater reduction in IFNβ MFI compared to classic CD68+ Macs and CD19s undergoing CB2R pretreatment and LPS/ATP stimulation (*p* < 0.05) (Figure 4A). Again, this matched a trend of reduction in IFNβ FoP for CD68+ Macs with CB2R treatment. No significant changes in IFNγ were observed upon CB2R treatment for LPS/ATP-stimulated amyopathic or classic DM PBMCs. However, there were trends of decreasing IFNγ positivity for LPS/ATP-stimulated amyopathic cDCs, a trend not observed in classic cDCs under the same conditions.

### 3.4. Both Amyopathic and Classic DM LPS-Stimulated PBMCs Show Little Response to CB2R Agonist Treatment

**Figure 5:**
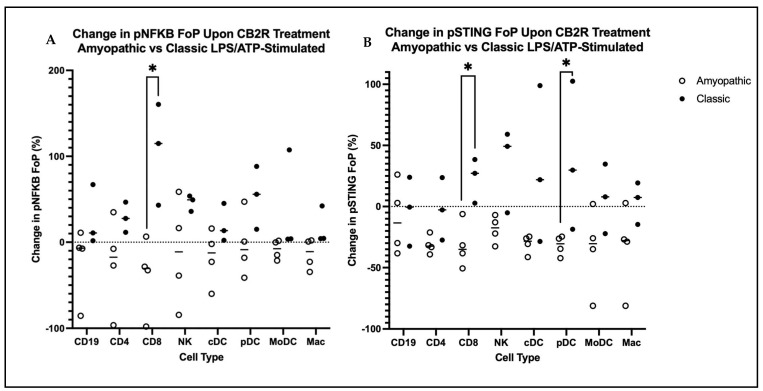
(**A**) Comparison of change in pNFKB FoP upon CB2R treatment for amyopathic and classic DM PBMCs that were stimulated with LPS/ATP to stimulate TLR4 and NLRP3 formation. LPS/ATP-stimulated amyopathic CD8 T cells showed a significant decrease in pNFKB FoP compared to classic LPS/ATP-stimulated CD8 T cells upon CB2R treatment (*p* < 0.05). (**B**) Comparison of change in pSTING FoP upon CB2R treatment for amyopathic and classic DM PBMCs that were stimulated with LPS/ATP to stimulate TLR4 and NLRP3 formation. LPS/ATP-stimulated amyopathic CD8 T cells and pDCs showed a significant decrease in pNFKB FoP compared to classic LPS/ATP-stimulated CD8 T cells and pDCs upon CB2R treatment (*p* < 0.05). * *p* < 0.05.

LPS stimulation is designed to target TLR4 activation only. After this stimulation, the only differences observed after CB2R treatment were amyopathic vs. classic CD19s, where amyopathic CD19s had significantly lower IFNβ MFI compared to classic CD19s. Otherwise, there were no discernible effects in reducing IFNβ, IFNγ, p65NFkB, or pSTING for both DM subtypes studied (Figure 4B). The percent reduction in p65NFkB FoP in LPS/ATP-stimulated amyopathic DM CD8+ T cells upon CB2R agonist pre-treatment was found to be significantly greater than in classic DM (*p* < 0.05) (Figure 5). Additionally, for LPS/ATP-stimulated MoDCs and Macs, the percent reduction in p65NFkB MFI upon CB2R treatment was found to be significantly greater in amyopathic DM compared to classic DM. Finally, the percent reduction in pSTING FoP in LPS/ATP-stimulated amyopathic DM CD8+ T cells and pDCs upon CB2R agonist pre-treatment was found to be significantly greater in amyopathic DM compared to classic DM (*p* < 0.05) (Figure 5). Regarding stimulation using dsRNA and dsDNA to target RIG1 and cGAS, respectively, little consistent stimulation was found across both DM subtypes; therefore, no effect could be found for CB2R treatment in reducing stimulation.

## 4. Discussion

A common finding in the various experiments performed here is the differences in reactions to CB2R treatment between amyopathic and classic DM PBMCs. This, coupled with our finding of different expression of the CB2R receptor itself on DM PBMCs from the two subtypes, suggests that there may be a connection between CB2R receptor expression and response to the CB2R agonist. The increased CB2R expression on amyopathic pDCs, cDCs, CD163- Macs, and CD163+ Macs, along with the overall higher sum of CB2R positivity in amyopathic DM compared to classic DM PBMCs, may be a major factor as to why JWH133 is more effective in reducing LPS/ATP-induced inflammation in amyopathic DM PBMCs compared to classic DM. This prompts the question of why amyopathic DM PBMCs express so much more CB2R than classics. Variation in CB2R expression is present in other diseases such as schizophrenia and multiple sclerosis [15,16]. PBMCs from schizophrenia patients were found to have higher CB2R expression compared to healthy controls, and higher CB2R expression is correlated with elevated severity of symptoms [15]. Because schizophrenia is thought to be 79% heritable, it suggests that elevated CB2R levels in these patients may be due to genetic factors [17]. Multiple sclerosis is thought to have some heritability, although this effect appears to be much weaker than in schizophrenia [18]. Nonetheless, multiple sclerosis PBMCs express higher CB2R levels than healthy controls, and when treated with pro-inflammatory cytokines IL1β, IL6, and TNFα, CB2R expression in both MS and control PBMCs is upregulated [19]. This effect is especially true with TNFα, and this is suspected to be the result of downstream NFkB activation [19]. There is upregulation of CB2R and increased IL1β, IL6 and TNFα mRNA in the blood of patients with MS compared with controls [19]. It is important to keep in mind that while proinflammatory cytokines upregulate CB2R, the increased CB2R in MS patients may be independent of these cytokines. These two diseases demonstrate how CB2R upregulation could be due to either genetic or local inflammatory conditions.

Whether upregulation is a result of genetic or environmental factors leading to certain cytokine conditions, the large difference in CB2R expression between amyopathic and classic DM is noteworthy. This, coupled with the fact that these DM subtypes are symptomatically distinct, suggests that the underlying mechanisms driving disease may be different between the two. Based on the effect of cytokines on CB2R expression in MS, characterizing the cytokine signature of amyopathic and classic DM may reveal details about whether the difference in CB2R expression is due to specific cytokines or other factors. Furthermore, the involvement of the NFkB inflammatory pathway is worth investigating for amyopathic vs. classic DM. As in MS, its activation is thought to be a contributing factor to upregulated CB2R expression in PBMCs. Total CB2R positivity was not the only difference in CB2R expression found between amyopathic and classic DM PBMCs. In decreasing order, the PBMCs with the highest CB2R positivity for amyopathic DM were pDCs, NKTs, cDCs, NK, CD163+ Macs, and CD163- Macs, while in classic DM they were NKs, NKTs, pDCs, CD19s, and cDCs. Of note is the increased relevance of CB2R in pDCs, cDCs, and Macs in amyopathic compared to the classic DM PBMCs. This may point to the possibility that these cells play a bigger role in regulating disease in amyopathic compared to classic DM, suggesting that a different inflammatory pathway that involves these cells is active in amyopathic DM. Ultimately, this is a question that requires further investigation.

It was expected that CB2R treatment of LPS-stimulated PBMCs would reduce inflammation. However, this was not found to be the case with both DM subtypes studied. A study from our lab investigating Spirulina as an herbal trigger of DM found that when TLR4 was blocked, the inflammatory effects of spirulina were ameliorated, suggesting its inflammatory mechanism was through TLR4 [3]. The mechanistic pathway supporting our initial expectation was quite straightforward, as LPS activation results in NFkB activation via TLR4, and CB2R activation is purported to reduce p65NFkB. This effect was not observed in the change in cytokine output or p65NFkB positivity in CB2R agonist-treated cells stimulated with LPS. Instead, we found CB2R activation effective in reducing NLRP3-driven inflammation in amyopathic DM PBMCs. It was observed that CB2R agonist pre-treatment of LPS-stimulated DM PBMCs showed a trend towards increased p65NFkB. It is possible that spirulina leads to priming through TLR4, and then a second inflammatory stimulant can induce NFkB-mediated NLRP3 and pro-IL1b expression.

One of the most noteworthy results from these experiments is the difference in the reaction of LPS/ATP-stimulated PBMCs to CB2R pre-treatment for amyopathic vs. classic DM PBMCs. The CD19s, Macs, MoDCs, CD8+ T Cells, and CD123+ pDCs of amyopathic DM showed a reduction in inflammatory markers, while no cell types of classic DM showed a reduction. Although further investigation into the specific pathways mediating this finding is necessary, the difference in CB2R expression between the two DM subtypes may be a major factor, as more CB2R expression could mean a stronger effect upon activation. A similar phenomenon was noted in previous studies where cells from whole blood that responded to Lenabasum expressed higher CB2R levels compared to non-responders [19].

Of note in the inflammatory markers that changed as a result of CB2R activation is the decrease in pSTING for LPS/ATP-stimulated amyopathic CD8+ T cells and CD123+ pDCs. Interestingly, pSTING is not directly stimulated by LPS/ATP or CB2R, so this points to this pathway being secondary to the initial stimulation. While it may be secondary in this case, the role of pSTING in DM pathogenesis should not be discounted, as STING activation has been implicated in inflammatory cells in the skin and necrotic atrophic DM muscle fibers [20]. Because of this, a treatment that reduces its activation, if only indirectly, is of great interest.

## 5. Conclusions

There are limitations to consider when interpreting the results. Participation from more patients from other institutions would make the presented results more impactful; however, gathering large numbers of DM patients for the study is quite challenging. Additionally, many of these patients were undergoing treatment, creating possible confounding variables. The results of this study prompted many more questions that have not been investigated yet. Characterizing the cytokine signature of amyopathic versus classic DM could help explain the differences in CB2R expression between them. Along these lines, genetic factors could be investigated to explain this difference in CB2R expression. pSTING is associated with inflammatory damage in DM, so an experiment determining the relative contribution of this pathway in DM could be enlightening.

A major takeaway from these conclusions is the theory that classic and amyopathic DM subtypes may have differences in their mechanisms driving disease. This is most apparent in the difference in CB2R expression, suggesting a greater bias towards p65NFkB activity for amyopathic DM and a difference in the response to CB2R agonist treatment for ameliorating LPS/ATP-induced stimulation. Additionally, the greater CB2R expression on amyopathic DM PBMCs provides a mechanistic explanation and more reason to use cannabinoids such as JWH133 or Lenabasum in treating clinical disease. These results highlight the idea that DM patients should have treatments specific to their subtype, as their inflammatory mechanisms may be different; however, more research is needed to explore this topic.

## Figures and Tables

**Table 1:** PBMC donor data.

Pt #	Date	DM Subtype	CDASI-A	Meds
1	5 January 2024	CADM	5	Took plaquenil briefly in past (uncertain of duration); currently on no meds
2	22 January 2024	Classic	13	Treated with topicals, plaquenil (6 months), methotrexate, imuran, thalidomide, dupixent in past, now on IVIG since March/2021.
3	9 February 2024	Classic	31	Currently on pred, IVIG, and MMF; previously was on benlysta, saphnelo, and plaquenil
4	16 February 2024	CADM	35	No previous/current meds besides topicals
5	1 March 2024	CADM	15	On plaquenil; no other past meds
6	18 March 2024	Classic	7	Treatment naïve
7	29 March 2024	CADM	16	No Previous/Current Meds Besides Topicals

## Data Availability

The patient PBMC datasets generated and analyzed during this study are available from the corresponding author on reasonable request.

## Supplementary Materials

The following supporting information can be downloaded at: https://www.mdpi.com/article/10.3390/biomedicines14061296/s1.

## Abbreviations

The following abbreviations are used in this manuscript:

DM	Dermatomyositis
CB2R	Cannabinoid type-2
IFN	Interferon
NLRP3	Nod-like receptor protein 3
TLR4	Toll-like receptor 4
PRR	Pattern recognition receptor
cGAS	Cyclic GMP-AMP synthase
p	Phospho
STING	Stimulator of interferon genes
IRF3	Interferon regulatory factor 3
TBK1	Tank binding kinase 1
NFkB	Nuclear factor kappa B
HC	Healthy control
EV	Extracellular vesicle
RIG-1	Retanoic acid-inducible gene 1
CD4	Helper T cell
CD8	Cytotoxic T cell
NK	Natural killer cell
NKT	Natural killer T cell
CD19	CD19+ B cells
MoDC	Monocyte-derived dendritic cell
M2	Macrophage 2
M1	Macrophage 1
cDC	Conventional dendritic cells
pDC	Plasmacytoid dendritic cells
iMs	Intermediate monocytes
CM	Classical monocytes
MMF	Mycophenolate
Classic	Dermatomyositis subtype
CADM	Clinically amyopathic dermatomyositis
CDASI-A	Cutaneous Dermatomyositis Disease Area and Severity Index-Activity

## References

[1] Robinson A.B., Reed A.M. (2011). Clinical Features, Pathogenesis and Treatment of Juvenile and Adult Dermatomyositis. Nat. Rev. Rheumatol..

[2] Concha J.S.S., Tarazi M., Kushner C.J., Gaffney R.G., Werth V.P. (2019). The Diagnosis and Classification of Amyopathic Dermatomyositis: A Historical Review and Assessment of Existing Criteria. Br. J. Dermatol..

[3] Bax C.E., Diaz D., Li Y., Vazquez T., Patel J., Grinnell M., Ravishankar A., Maddukuri S., Keyes E., Yan D. (2023). Herbal Supplement Spirulina Stimulates Inflammatory Cytokine Production in Patients with Dermatomyositis In Vitro. iScience.

[4] Patel J., Maddukuri S., Li Y., Bax C., Werth V.P. (2021). Highly Multiplexed Mass Cytometry Identifies the Immunophenotype in the Skin of Dermatomyositis. J. Investig. Dermatol..

[5] Li Y., Bax C., Patel J., Vazquez T., Ravishankar A., Bashir M.M., Grinnell M., Diaz D., Werth V.P. (2021). Plasma-Derived DNA Containing-Extracellular Vesicles Induce STING-Mediated Proinflammatory Responses in Dermatomyositis. Theranostics.

[6] Suárez-Calvet X., Gallardo E., Pinal-Fernandez I., De Luna N., Lleixà C., Díaz-Manera J., Rojas-García R., Castellví I., Martínez M.A., Grau J.M. (2017). RIG-I Expression in Perifascicular Myofibers Is a Reliable Biomarker of Dermatomyositis. Arthritis Res. Ther..

[7] Yin X., Han G.-C., Jiang X.-W., Shi Q., Pu C.-Q. (2016). Increased Expression of the NOD-like Receptor Family, Pyrin Domain Containing 3 Inflammasome in Dermatomyositis and Polymyositis Is a Potential Contributor to Their Pathogenesis. Chin. Med. J..

[8] Wong D., Kea B., Pesich R., Higgs B.W., Zhu W., Brown P., Yao Y., Fiorentino D. (2012). Interferon and Biologic Signatures in Dermatomyositis Skin: Specificity and Heterogeneity across Diseases. PLoS ONE.

[9] Huard C., Gullà S.V., Bennett D.V., Coyle A.J., Vleugels R.A., Greenberg S.A. (2017). Correlation of Cutaneous Disease Activity with Type 1 Interferon Gene Signature and Interferon β in Dermatomyositis. Br. J. Dermatol..

[10] Fiorentino D., Mangold A.R., Werth V.P., Christopher-Stine L., Femia A., Chu M., Musiek A.C.M., Sluzevich J.C., Graham L.V., Fernandez A.P. (2025). Efficacy, Safety, and Target Engagement of Dazukibart, an IFNβ Specific Monoclonal Antibody, in Adults with Dermatomyositis: A Multicentre, Double-Blind, Randomised, Placebo-Controlled, Phase 2 Trial. Lancet.

[11] Werth V.P., Hejazi E., Pena S.M., Haber J., Zeidi M., Reddy N., Okawa J., Feng R., Bashir M.M., Gebre K. (2022). Safety and Efficacy of Lenabasum, a Cannabinoid Receptor Type 2 Agonist, in Patients with Dermatomyositis with Refractory Skin Disease: A Randomized Clinical Trial. J. Investig. Dermatol..

[12] Werth V., White B., Dgetluck N., Hally K., Constantine S., Aggarwal R., Fiorentino D., Lundberg I.E., Oddis C.V. (2022). OP0162 Efficacy and Safety of Lenabasum in the Phase 3 Determine Trial in Dermatomyositis. Ann. Rheum. Dis..

[13] Kodali N., Diaz D., Dhiman R., Vazquez T., Feng R., Patel J., Dan J., Sprow G., Kleitsch J., Sharma M. (2025). Diverse Lenabasum Pathway Activation in Dermatomyositis Patients’ Blood. Sci. Rep..

[14] Diaz D., Vazquez T., Kodali N., Bashir M.M., Grinnell M., Dhiman R., Keyes E., Dan J., Kleitsch J., Stone C.J. (2026). Lenabasum, a Cannabinoid Type 2 Receptor Agonist, Exerts Anti-Inflammatory Effects in Dermatomyositis. J. Investig. Dermatol..

[15] Chase K.A., Feiner B., Rosen C., Gavin D.P., Sharma R.P. (2016). Characterization of Peripheral Cannabinoid Receptor Expression and Clinical Correlates in Schizophrenia. Psychiatry Res..

[16] Jean-Gilles L., Feng S., Tench C.R., Chapman V., Kendall D.A., Barrett D.A., Constantinescu C.S. (2009). Plasma Endocannabinoid Levels in Multiple Sclerosis. J. Neurol. Sci..

[17] Hilker R., Helenius D., Fagerlund B., Skytthe A., Christensen K., Werge T.M., Nordentoft M., Glenthøj B. (2018). Heritability of Schizophrenia and Schizophrenia Spectrum Based on the Nationwide Danish Twin Register. Biol. Psychiatry.

[18] Patsopoulos N.A. (2018). Genetics of Multiple Sclerosis: An Overview and New Directions. Cold Spring Harb. Perspect. Med..

[19] Jean-Gilles L., Braitch M., Latif M.L., Aram J., Fahey A.J., Edwards L.J., Robins R.A., Tanasescu R., Tighe P.J., Gran B. (2015). Effects of Pro-Inflammatory Cytokines on Cannabinoid CB1 and CB2 Receptors in Immune Cells. Acta Physiol..

[20] Zhou M., Cheng X., Zhu W., Jiang J., Zhu S., Wu X., Liu M., Fang Q. (2022). Activation of cGAS-STING Pathway—A Possible Cause of Myofiber Atrophy/Necrosis in Dermatomyositis and Immune-Mediated Necrotizing Myopathy. J. Clin. Lab. Anal..

