# Supplementary material for: The Effects of CB2R Activation on Inflammatory Pathways in Dermatomyositis
Source: Biomedicines. 2026 Jun 7;14(6):1296. doi: 10.3390/biomedicines14061296 (PMC13296682; doi:10.3390/biomedicines14061296)

Figure S1

LPS/ATP Stimulated Amyopathic vs Classic Change in NFKB FoP

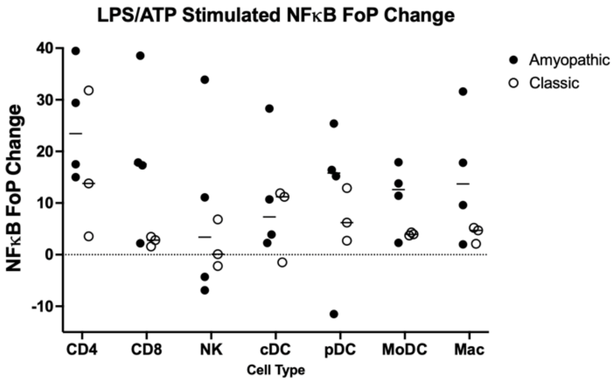

LPS/ATP Stimulated Amyopathic vs Classic Change in NFKB MFI

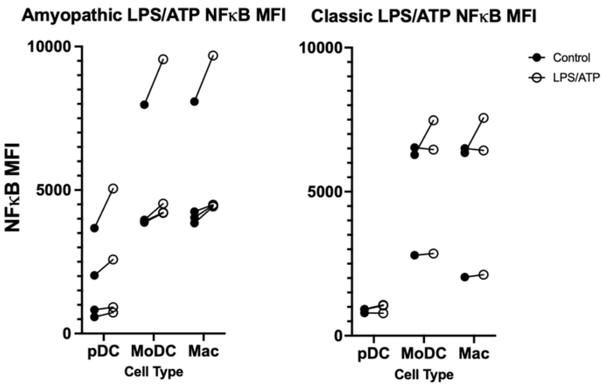

Figure S2

dsDNA Stimulated Amyopathic vs Classic Change in NFKB FoP

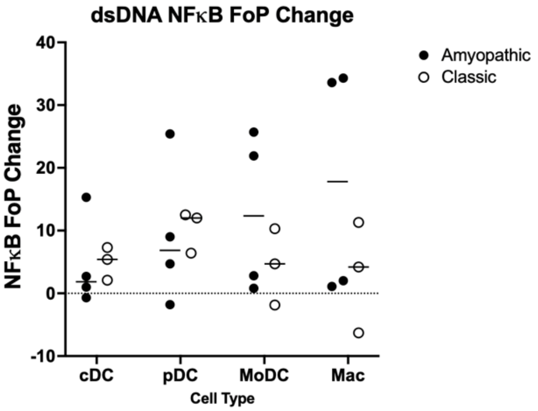

Figure S3

LPS Stimulated Amyopathic vs Classic Change in NFKB FoP

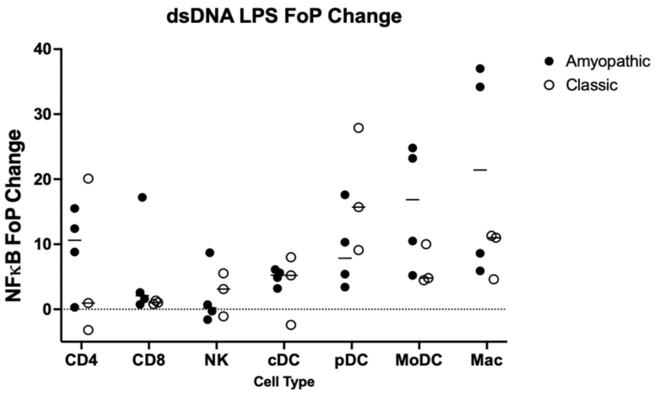

Figure S4

dsRNA Stimulated Amyopathic vs Classic
Change in pSTING FoP

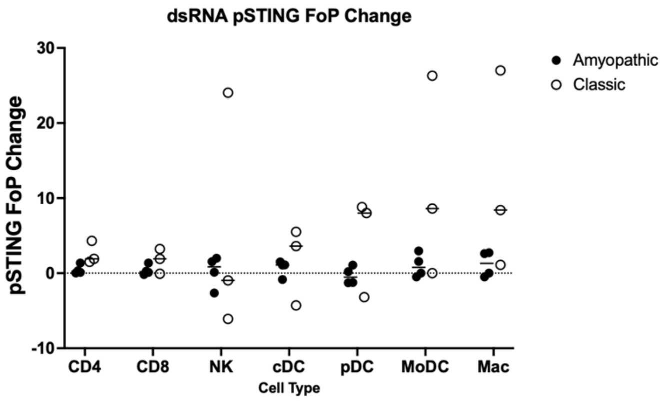

Figure S5

LPS Stimulated Amyopathic vs Classic
Change in pSTING FoP

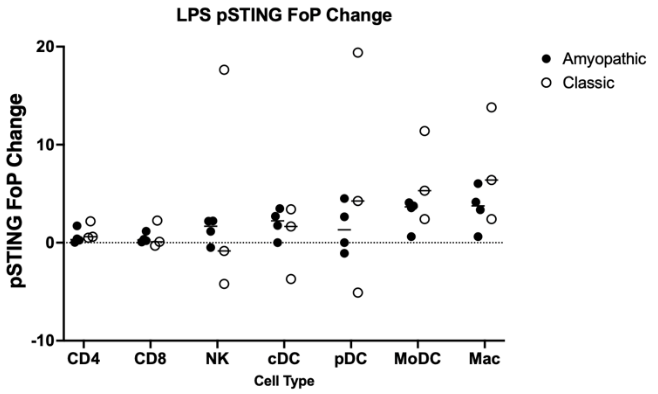

Figure S6

LPS/ATP Stimulated Amyopathic vs Classic
Change in pSTING FoP

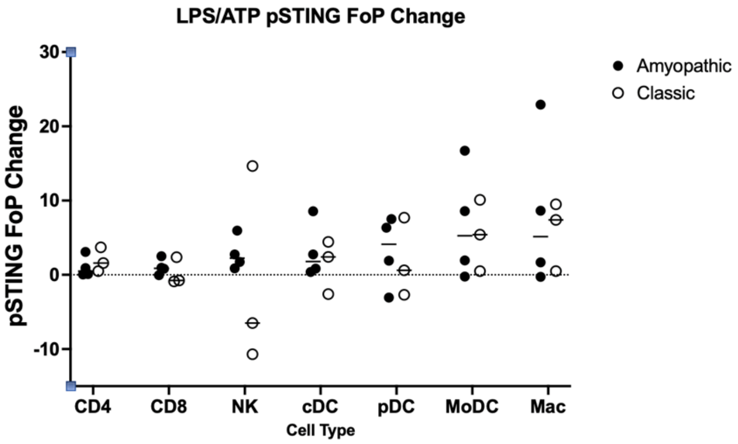

Figure S7

CB2R Treated L/A Stimulated Amyopathic
vs Classic NFKB % Change MFI

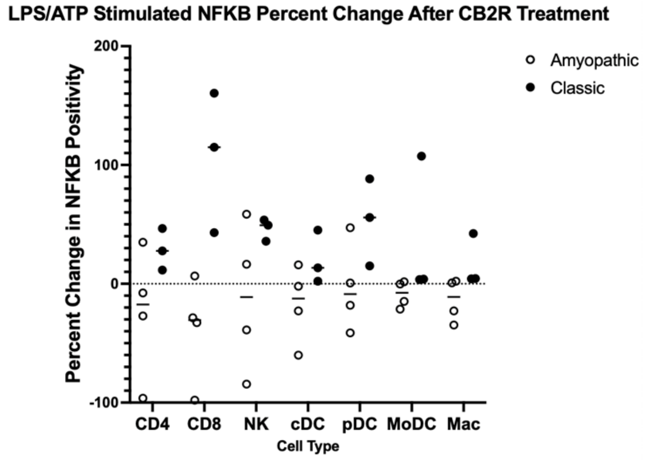

Figure S8

CB2R Treated L/A Stimulated Amyopathic
vs Classic pSTING % Change MFI

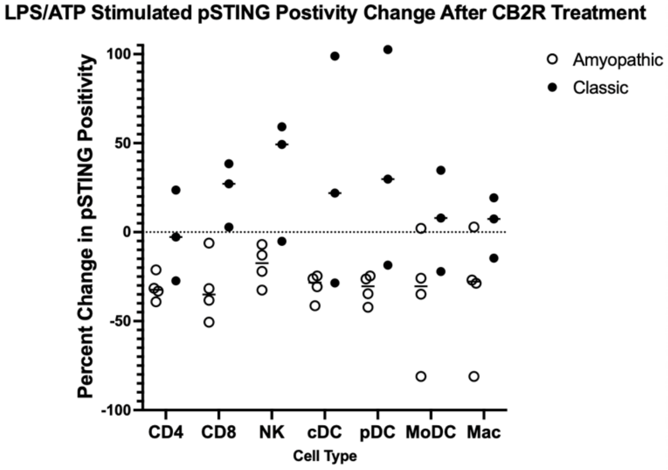

Supplement: Supplementary file 1 [file biomedicines-14-01296-s001.zip › biomedicines-4204577-supplementary.pdf]
